# Understanding End-User Perspectives of Mobile Pulmonary Rehabilitation (mPR): Cross-Sectional Survey and Interviews

**DOI:** 10.2196/15466

**Published:** 2019-12-20

**Authors:** Rosie Dobson, Pauline Herbst, Sarah Candy, Tamzin Brott, Jeffrey Garrett, Gayl Humphrey, Julie Reeve, Merryn Tawhai, Denise Taylor, Jim Warren, Robyn Whittaker

**Affiliations:** 1 National Institute for Health Innovation University of Auckland Auckland New Zealand; 2 Waitemata District Health Board Auckland New Zealand; 3 Counties Manukau District Health Board Auckland New Zealand; 4 School of Clinical Sciences Auckland University of Technology Auckland New Zealand; 5 Auckland Bioengineering Institute University of Auckland Auckland New Zealand; 6 School of Computer Science University of Auckland Auckland New Zealand

**Keywords:** mHealth, rehabilitation, COPD

## Abstract

**Background:**

Pulmonary rehabilitation (PR) is an effective intervention for the management of people with chronic respiratory diseases, but the uptake of and adherence to PR programs is low. There is potential for mobile health (mHealth) to provide an alternative modality for the delivery of PR, overcoming many of the barriers contributing to poor attendance to current services.

**Objective:**

The objective of this study was to understand the needs, preferences, and priorities of end users for the development of an adaptive mobile PR (mPR) support program.

**Methods:**

A mixed methods (qualitative and quantitative) approach was used to assess the needs, preferences, and priorities of the end users (ie, patients with chronic respiratory disorders) and key stakeholders (ie, clinicians working with patients with chronic respiratory disorders and running PR). The formative studies included the following: (1) a survey to understand the preferences and priorities of patients for PR and how mobile technology could be used to provide PR support, (2) ethnographic semistructured interviews with patients with chronic respiratory disorders to gain perspectives on their understanding of their health and potential features that could be included in an mPR program, and (3) key informant interviews with health care providers to understand the needs, preferences, and priorities for the development of an mPR support program.

**Results:**

Across all formative studies (patient survey, n=30; patient interviews, n=8; and key stakeholder interviews, n=8), the participants were positive about the idea of an mPR program but raised concerns related to digital literacy and confidence in using technology, access to technology, and loss of social support currently gained from traditional programs. Key stakeholders highlighted the need for patient safety to be maintained and ensuring appropriate programs for different groups within the population. Finding a balance between ensuring safety and maximizing access was seen to be essential in the success of an mPR program.

**Conclusions:**

These formative studies found high interest in mHealth-based PR intervention and detailed the potential for an mPR program to overcome current barriers to accessing traditional PR programs. Key considerations and features were identified, including the importance of technology access and digital literacy being considered in utilizing technology with this population.

## Introduction

Chronic obstructive pulmonary disorder (COPD), an umbrella term for a range of debilitating respiratory diseases [1], is the fourth leading cause of mortality worldwide [2]. In New Zealand (NZ), COPD affects approximately 14% of adults aged >40 years [3]. Maori, the NZ indigenous population, as well as ethnic minority groups and those from socioeconomically deprived groups are disproportionately affected by COPD with higher prevalence rates and hospitalizations and are more likely to die from the condition [2,3].

One of the most effective interventions for COPD is pulmonary rehabilitation (PR), an evidence-based, interdisciplinary intervention that is a key component in the management of people with respiratory diseases [4]. PR is a formalized structured program comprising, but not limited to, exercise training, education, and behavior change, and it is designed to improve a patient’s physical and psychological health and encourage engagement with health-enhancing behaviors [5]. PR is an individually tailored intervention based on thorough patient assessment, which is typically delivered in group programs in hospital or community settings. PR has been clearly demonstrated to improve breathlessness and health-related quality of life and reduce hospital admissions for exacerbations of COPD [4,6]. Clinical guidelines strongly recommend the uptake of PR by all patients with COPD, particularly following hospital admissions [7]. Despite this, and PR programs being available across nearly all regions of NZ, the uptake of, and adherence to, PR programs in NZ is poor [8]. It was estimated that in 2012, <1% of all patients with COPD were participating in PR in NZ [8]. Poor attendance and adherence to PR programs is common internationally. Previous literature has suggested that this is because of transportation, lack of perceived benefit, depression, and the interruption to the patient’s daily routines [9-11]. In addition, many patients experience significant barriers to accessing PR services, especially those living in rural areas and where transportation to a central service may be unaffordable or unavailable. Home-based PR programs have been shown to overcome some of these access barriers for people with chronic respiratory diseases [12-14].

There is potential for mobile health (mHealth) to provide an alternative modality for the delivery of PR, overcoming many of the barriers contributing to poor attendance to current services. By utilizing mobile technology, PR can be made available to people within their everyday lives at times and places most suitable to the patient, removing the barriers associated with transport, timing, and location. There is a wealth of evidence for the use of mobile technology to deliver health interventions to people with chronic conditions including the delivery of rehabilitation interventions, self-management support programs, behavior change interventions, and supportive care [15-18]. Not only does mHealth allow for individually tailored interventions to be easily delivered in a cost-effective way but also it has potential for interventions to be adapted over time as individual needs and characteristics change [19].

When developing new mHealth tools, engagement with end users in the design is essential. By incorporating the perspectives of end users, it ensures the intervention will meet the population need and enables it to be tailored to specific cultural needs, contexts, and levels of technology access [20]. When end users are not considered in the design, it can contribute to poor uptake and use of tools [21]. Furthermore, it is important that formative research, including adequate description of the population context, is reported in the development of new mHealth interventions [22]. Formative research provides the basis for designing tools to meet user needs within system constraints and the local context.

This study aimed to understand the needs, preferences, and priorities of end users for the development of a mobile PR (mPR) support program. Specifically, it aimed to understand (1) the preferences and priorities of patients for PR and how mobile technology could be used to provide PR support and (2) the needs, preferences, and priorities of health care providers including physiotherapists, respiratory physicians, primary care nurses, and general practitioners (GPs).

## Methods

### Study Design

This cross-sectional study utilized a mixed method (qualitative and quantitative) approach incorporating surveys and interviews to assess the needs, preferences, and priorities of end users and key stakeholders. The study was split into 3 parts—(1) patient survey, (2) patient interviews, and (3) key stakeholder interviews—and was conducted between July and December 2018.

### Ethical Approval

Ethics approval was obtained from the NZ Health and Disability Ethics Committee (18/NTA/105). Research approval from each District Health Board (DHB) from which patients were recruited was also obtained. Written informed consent was obtained from survey and interview participants before their participation in the studies.

### Part 1: Patient Survey

#### Inclusion Criteria

Inclusion criteria were adults with chronic respiratory disease who would be eligible for PR, able to read and understand English, and provide informed consent.

#### Recruitment and Procedures

Potential participants were identified by clinicians at 2 DHBs in the Auckland region, NZ, through respiratory outpatient clinics and inpatient services. Eligible participants were given a letter about the study, the information sheet, and consent form. By selecting patients from this defined subgroup, we were more likely to define patients who had either been invited to attend or had attended a rehabilitation program. Those interested in participating provided informed consent before completing the survey via 1 of 3 methods: (1) on the web via the study website, (2) over the phone with a member of the research team, or (3) on paper. Surveys completed by phone or on paper were entered into the Web-based version of the survey by the researcher.

#### Survey Design

The survey comprised 4 parts and contained both closed- and open-ended questions to allow participants to elaborate on their answers: (1) your health including diagnosis, attendance at PR, and barriers to attendance and completion of PR; (2) technology access, including current use and access to technology and devices; (3) technology and pulmonary support, including perceptions of mPR, preferences for technology-based support, and perceived barriers and benefits to technology-based pulmonary support; and (4) demographics, specifically age group, gender, and ethnicity.

The survey was designed in paper format and then uploaded into REDCap software (v8.5.0). It was pretested by the research team and a selection of patients before finalization.

### Part 2: Patient Interviews

#### Inclusion Criteria

Survey participants who had consented to be contacted for future research following completion of the survey were eligible for inclusion in an interview.

#### Procedures

Patients who completed part 1 of this study and identified that they were happy to be contacted about further research were contacted to invite them to take part in an interview. Participants provided informed consent to participate as well as consent to access medical records related to their eligibility for PR. Interviews were conducted in person, or over the phone, by a trained interviewer (PH) at a time and location convenient to the participant. If needed, interviews were split over multiple sessions to reduce burden for participants. Notes were taken by the researcher during the interviews, and the interviews were recorded to supplement the notes. At the end of the interview process, the participants were offered a NZ $20.00 (approximately US $13.00 or €11.50) voucher for their time.

#### Interview Design

Ethnographic, semistructured interviews were undertaken. The interviews were designed to explore in-depth beliefs and perceptions of chronic respiratory disorders and their treatments, perspectives on potential features of an mPR intervention, and patient understanding of their health.

### Part 3: Key Stakeholder Interviews

#### Inclusion Criteria

Inclusion criteria were clinicians managing patients with COPD and other chronic pulmonary disorders (eg, bronchiectasis and interstitial lung disorders), able to read and understand English, and able to provide informed consent.

#### Procedures

Potential participants were identified through respiratory and PR services by coinvestigators RW, PH, SC, and JR and invited to take part via email. Interviews were conducted in person or via phone at the participant’s preference by a trained interviewer (RD) and were between 30 min and 1 hour in duration. Notes were taken by the interviewer, and the interviews were recorded to supplement the notes.

#### Interview Design

Interviews were semistructured and designed to cover the current use of technology in care of people with chronic respiratory conditions, experiences with PR, and perceptions of mPR including enablers and barriers.

### Statistical Analysis

Quantitative data from the survey were analyzed and summarized using descriptive quantitative analyses including means, standard deviations, and proportions. Qualitative comments were analyzed using a simple, general inductive thematic approach to identify common themes and meanings from the data. Only completed surveys were included in the analysis. Prioritized ethnicity was used as recommended by the NZ Ministry of Health for the reporting of ethnicity data; only 1 of the ethnic categories nominated by the participant was used according to a predetermined hierarchy (Maori, Pacific Islander, Asian, European, and other ethnic groups, in order of prioritization).

## Results

### Part 1: Patient Survey

There were 34 entries to the survey site from which 30 people consented to take part and completed the survey. Slightly more than half of the sample were male (17/30, 57%), and the majority were aged >65 years (22/30, 73%; Table 1). Over one-third of the participants identified as Maori or Pacific Islander (11/30, 37%). Approximately half the sample reported that they had been diagnosed with COPD (16/30, 53%). A total of 7 (7/30, 23%) participants reported that they were unsure, or did not know, their diagnosis.

**Table 1:** Demographic and clinical characteristics (N=30).

Characteristics		Participants, n (%)
**Gender**		
	Male	17 (57)
	Female	12 (40)
	Did not answer	1 (3)
**Ethnicity**		
	New Zealand European	17 (57)
	Maori	7 (23)
	Pacific	4 (13)
	Asian	0 (0)
	Other	1 (3)
	Did not answer	1 (3)
**Age (years)**		
	<45	0 (0)
	45-54	3 (10)
	55-64	4 (13)
	65-74	10 (33)
	75-84	12 (40)
	>85	0 (0)
	Did not answer	1 (3)
**Patient-reported diagnosis**		
	Chronic obstructive pulmonary disorder	16 (53)
	Emphysema	3 (10)
	Bronchitis	1 (3)
	Pulmonary fibrosis	1 (3)
	Bronchiectasis	1 (3)
	Asbestosis	1 (3)
	Do not know or unsure	7 (23)

#### Pulmonary Rehabilitation

Participants were asked if they had attended a PR program. Almost half (14/30, 47%) reported that they had completed PR or were intending to complete it in the future. Nearly a quarter of participants (7/30, 23%) had started a PR program but had not completed it. The reasons for not completing the program included issues related to location and transport (n=2), timing of the sessions (n=2), being hospitalized (n=2), and other commitments (n=1). A total of 3 participants (3/30, 10%) were offered PR but did not attend because of transport (n=2) or other commitments (eg, having to care for grandchildren; n=1). The remaining 6 (6/30, 20%) participants reported that they had not been offered PR or did not remember being offered PR.

#### Access to Technology

Participants were asked about their access to digital devices (eg, mobile phones, tablets, computers, and sensors) for personal use, with all but 4 (4/30, 13%) participants reporting having access to a mobile phone. Of those who had a mobile phone, the majority had a smartphone (20/26, 77%) and the remainder (6/26, 23%) a mobile phone without internet capability. Of those that had a smartphone, only 60% (12/20) reported having access to the internet on the smartphone all the time. A total of 5 (5/20, 25%) had access to the internet on the phone sometimes, 2 (2/20, 10%) not at all, and 1 (1/20, 5%) did not answer.

One-third of the sample (10/30, 33%) reported having access to a tablet for personal use, 14 (14/30, 47%) a computer (ie, laptop or desktop), 2 (2/30, 7%) a Fitbit or other fitness tracking device, and only 1 participant reported access to no devices for personal use. A total of 23 (23/30, 77%) participants reported access to internet at home, and an additional 3 (3/30, 10%) reported they sometimes had access to internet at home. There were 4 (4/30, 13%) that had no access to the internet at home.

In relation to the use of technology-based devices and tools to manage health, there was only 1 participant (1/30, 3%) that reported using an app, 1 (1/30, 3%) who reported using a smart watch, 3 (3/30, 10%) used a fitness tracking device, and 4 (4/30, 13%) a peak flow meter. A total of 20 (20/30, 67%) participants reported no use of these tools to manage their health.

#### Technology and Respiratory Health Support

Participants were asked about their perceptions of a technology/mobile phone program that would allow people to receive PR support at home. The majority (23/30, 77%) reported that they liked this idea, and 7 (7/30, 23%) did not like the idea. The proportion of those that liked the idea was highest in Maori participants (6/7, 86%) compared with NZ European (13/17, 77%) or Pacific (2/4, 50%). Those that liked the idea (n=23) were asked what they liked about the idea. The most common theme was around the access at home meaning no need to travel (n=10), and others reported the cost reduction related to parking and travel (n=3), being able to access the program wherever and whenever (n=1), less embarrassment within the home (n=1), and that family would be able to help and be involved (n=1):

Privacy—don’t have to be embarrassed.

45-54-year-old male, ID number 27

Would make it easier as would not have to travel in bad weather.

75-84-year-old male, ID number 8

The convenience. At the moment, travel outside the home is quite onerous for me.

65-74-year-old male, ID number 9

Family would be able to help Dad to do this at home.

75-84-year-old male, ID number 25

Would have saved me some trips... Transport is hard for people, don't have a car.

45-54-year-old male, ID number 27

There were 5 participants whose responses were moderately supportive of it being a good idea and 3 who did not answer the question. In addition, 3 participants identified concerns around the proposed idea potentially resulting in less social contact and less access to health care professionals:

Sounds good although I do like meeting other people.

65-74-year-old female, ID number 33

But it is good to meet other people and learn from others.

65-74-year-old female, ID number 22

Those that did not like the idea (n=7) were asked the reason, with the most common theme being that they found technology too difficult/hard (n=3). Other themes included not having internet (n=1), wanting the company/social aspect of an in-person group program (n=1), or preferring to go to the hospital (n= 1; there was 1 participant who did not provide a reason):

Too hard to use computer.

75-84-year-old male, ID number 18

No internet at home.

55-64-year-old male, ID number 19

I would feel isolated, when you are older you need company.

75-84-year-old female, ID number 21

Participants were asked about the features they thought would be helpful to include in the proposed program. Of those that liked the proposed idea and who responded to the question (n=22), 19 (19/22, 86%) selected tips and suggestions for managing their breathing, 17 (17/22, 77%) information and education about their condition, 12 (12/22, 54%) access to their health information, 11 (11/22, 50%) tracking of their health data, and 9 (9/22, 41%) motivational and support messages. A total of 23 (23/30, 77%) reported that they would consider wearing a sensor (eg, a Fitbit or activity tracker) as part of an mPR program, and 22 (22/30, 73%) reported that they would want the program to be linked into their existing health record and information so that the program could be tailored to their individual health condition and treatment.

Participants were asked to identify the barriers to using the proposed mPR program (Table 2). The most common barrier was concern about not being comfortable or confident using technology.

When asked about the perceived benefits of using technology to support people with health conditions in the community, the majority (25/29, 86%) identified the convenience of having access at any time or anywhere as a benefit. There were 15 (15/29, 52%) participants who identified the benefit of being able to involve/include family/whanau, and 4 (4/29, 14%) identified feeling more comfortable not being in the group or clinical environment.

**Table 2:** Barriers to using a mobile pulmonary rehabilitation program (N=30).

Barriers	Participants, n (%)
No barriers	6 (20)
No access to technology	5 (17)
Do not feel comfortable using technology	11 (37)
Security concerns regarding my health information	3 (10)
Not interested in this type of support	3 (10)
Could increase worry and anxiety	1 (3)
Would not be useful if not translated	1 (3)

### Part 2: Patient Interviews

A total of 8 patient interviews were conducted. Participants included 2 women (2/8, 25%) and 6 men (6/8, 75%), with 2 of the 8 (2/8, 25%) individuals identifying as Maori and the remaining (6/8, 75%) as NZ European. The majority were aged 75 to 84 years (4/8, 50%), 2 (2/8, 25%) aged 65 to 74 years, and the remaining (2/8, 25%) aged 45 to 54 years. Participants were registered across 2 DHBs and interviewed at home (2/8, 25%), in the hospital (5/8, 63%), or via phone (1/8, 13%). Analysis of the interview data is summarized in 4 major themes.

#### Condition and Health

Participants identified themselves as having COPD, emphysema, and asbestosis, but only 6 participants were able to clearly describe their diagnosis and treatment. Diagnosis was reported as being a shock to receive; however, this shock did not necessarily change behavior:

Well I didn’t know much about it, so it didn’t make much of a difference to me. I continued smoking, didn’t I?

75-84-year-old male, ID number 4

I knew from the day he said that, that things were not so good for me.

75-84-year-old male, ID number 6

Participants described COPD affecting all facets of their lives negatively. Despite this, all expressed some form of resilience and stoicism during the interviews:

It affects everything: social, mental, physical.

75-84-year-old female, ID number 5

It can't be cured you just have to live with it and get on with life and do what you've got to do.

75-84-year-old male, ID number 2

I’m pretty humble with it. I’ve lived a pretty good life.

45-54-year-old male, ID number 3

Only 2 participants showed some degree of understanding of their test results, and the remaining participants did not understand the results of their tests; they knew what their diagnosis was but found the presentation of the test results difficult to comprehend. All participants struggled to remember which tests had been conducted and at which point in their illness trajectory:

Honestly, I just go when I am told to go and have it done, and that’s it.

75-84-year-old female, ID number 5

Of the 8 participants, 7 were ex-smokers and acknowledged this as a contributing factor to their current respiratory condition; however, 5 offered additional environmental factors as contributors including asbestos and work environments.

#### Pulmonary Rehabilitation

All patients interviewed had some experience of hospital-based PR programs. A total of 2 had completed programs within the past 3 years, 2 had started but stopped indefinitely because of hospitalization, 3 were in the process of completing an 8-week program, and 1 had tried a program over a decade previously. All but 1 reported enjoying the programs:

I wish I had done it earlier.

75-84-year-old male, ID number 2

I love it. I wish they had it 12 months of the year.

65-74-year-old female, ID number 7

Yeah, I enjoy the exercise. It's just a matter of getting your mind in the right place. I get up and I do it and I feel a lot better afterwards.

65-74-year-old male, ID number 1

Reasons for this included socializing during the training, being able to compare progress and daily lived experiences with others with a similar condition, compassionate and dedicated staff who were known to the participants, and both a measurable and perceived improvement in physical ability. However, a participant reported anxiety and an increased sense of vulnerability after he was transferred to community support, following completion of the hospital-based PR program, arising from inconsistency in times and scheduling, different staff members at each visit, and a decreased sense of support.

#### Technology-Based Pulmonary Rehabilitation

All but 1 of the participants (7/8, 88%) described that they preferred to be offered a PR program in person at the moment of diagnosis by a medical professional they trusted and had an existing relationship with, rather than when in hospital with an exacerbation:

When you’re really sick and they come at you, like the physio comes, and this one comes, and that one comes, and you just feel like being left alone, I felt, just leave me alone sort of thing... maybe after being first diagnosed.

75-84-year-old female, ID number 5

The main barrier to mPR was a perceived incompetence with technology and a fear that this would be difficult to work with; however, 6 participants appeared to be confident texting during interviews, and 4 talked about using Facebook to communicate with family. A participant enjoyed using health apps to measure aspects of his health.

Feature suggestions for mPR included knowing which part of the body exercises were targeting, with the most ambitious proposals suggesting:

I think you should give them every feature that you can and maybe give them the option to choose... incorporating videos of people doing it and how they started and then how they finished... a motivational start, and then a talk and showing “look at me, now I can run two kilometers.

45-54-year-old male, ID number 3

#### Imaging

During the interview, participants were shown a model of the lung [23,24] and an interactive website [25]. The subsequent discussion revealed the underlying power of visual imagery to reveal the *truth* of diagnosis, with patients reporting that they struggled to accept their diagnosis until they saw an image and that seemed more *real* than paper, which they saw as a tool for the medical professional:

I didn’t get how bad my lungs were until I saw it.

45-54-year-old male, ID number 3

She said “have you ever seen your lung?” and showed me this x-ray and I thought, “whoa.”

75-84-year-old male, ID number 2

A participant said she had never been shown an x-ray or image of her lungs and insisted that this would have caused her to stop smoking earlier.

### Part 3: Key Stakeholder Interviews

A total of 8 key stakeholder interviews were completed. Key stakeholders included 2 doctors (ie, GP and respiratory specialist), 2 nurses, 3 physiotherapists, and 1 health psychologist. Participants worked across either predominantly urban (5/8, 63%) or predominantly rural (3/8, 37%) populations. A total of 5 (5/8, 63%) participants were directly involved in the delivery of PR, whereas the remaining 3 (3/8, 37%) were involved in referring patients to PR services.

When asked about their perceptions of patients’ understanding of COPD, all acknowledged that it was generally poor. A total of 3 participants acknowledged that understanding varied, and those that had attended PR or sought health information through the internet had a better understanding. Common tools for explaining COPD to patients included handouts and pamphlets (n=3), Web resources (ie, websites and YouTube videos; n=2), drawings and models (n=2), and patient scans (n=1).

#### Mobile Pulmonary Rehabilitation

All participants thought that a technology- or mobile-based PR program was a good idea, particularly for overcoming the barriers their patients currently experienced to attending PR. However, the majority of the participants also raised concerns that an mPR program would lack the social element and not be suitable for some groups such as older patients or those with limited confidence and access to technology.

Participants were asked how they felt an mPR program would fit into current models of care. Responses could be grouped into 3 main categories: (1) an alternative service to increase the access to PR for those that were not able to attend current services, (2) a maintenance program following traditional in-person PR programs, and (3) a combination/mixed model of care where patients used an mPR program to complement the in-person program. All participants reported that an mPR program should include the following components: (1) education, (2) exercise information, (3) motivation and support, (4) the ability to view personal health information/data, and (4) monitoring of health behaviors.

Other components identified by the participants for inclusion in an mPR program included health psychology content, medication reminders, personalized action plans, step-by-step videos, social features, sharing of information between patient and health care professional, and general self-care information. Participants also identified that it would be essential for a program to be both individually and culturally tailored.

When asked about the different technologies the potential program should utilize, many different technologies were identified including sensors, smart inhalers, apps, and text messages. But consistent across all participants was that the technologies used needed to consider differing access to technology, devices, and data, as well as confidence with using technology:

Important to use mobile phone as a lot of people won't have anything else.

ID number 1

An app would be a good way of doing it... Sensors are useful for helping patients to see progress to goals.

ID number 2

Text messages are easy for patients to get, older patients to get, minority populations do not tend to have a lot of money on their phones.

ID number 3

A lot of people don’t have internet at all. We text or phone them, they aren’t able to text, call as they have no money on their phones. Some people don’t even have mobile coverage...

ID number 6

We already use SMS, people are used to getting text messages.

ID number 7

There were conflicting views from participants around how an mPR program should be accessed. A participant felt that referrals should be through the same avenues as current services to ensure safety was prioritized, and only people who were appropriate were accessing the program. Others felt that in addition to clinician referrals, patients should be able to access it directly to reduce barriers to access. There was only 1 participant who felt that clinicians should not be involved in referring to the program at all because of concerns that the clinician would need to then provide technical support:

Referred [by a clinician] is best rather than self-referral, ensures that the info will be right for that patient. Same criteria as current groups.

ID number 1

Shouldn’t put up a barrier of the clinician referring them. Especially if they can’t afford to see their GP.

ID number 2

Anyone should be able to have access to it but there needs to be some sort of way of knowing people are safe with it, that they are safe to exercise.

ID number 4

Patients should be able to access it themselves. Health care professionals could promote it but don’t want to become IT support.

ID number 5

Finally, participants were asked about the potential barriers/downsides to an mPR program. All but 1 participant (7/8, 88%) reported concerns relating to the digital divide, including access to technology and the confidence to use it. A full list of barriers identified can be seen in Table 3.

**Table 3:** Barriers to using a mobile pulmonary rehabilitation program (N=8).

Barriers	Participants, n (%)
Digital divide	7 (88)
The lack of the social/group environment	6 (75)
Lack of relationship between clinician and patient	4 (50)
Safety for patients not being supervised	2 (25)
Patient compliance to the program	2 (25)
Health care professional digital literacy	1 (13)
Successful marketing and promotion of the program	1 (13)
Patient access to exercise equipment	1 (13)

## Discussion

### Principal Findings

This study aimed to identify the needs, preferences, and priorities of end users in the development of an adaptive mPR support program. A survey of patients, together with interviews of patients and key stakeholders, found a common interest in an mPR program. The potential for mPR overcoming barriers to accessing traditional PR programs was highlighted. These findings are consistent with previous research reporting high acceptability of digital health tools in patients with chronic respiratory conditions [26-28].

This formative study has identified important aspects of our target audience and their diversity of needs. Some have low access to digital technology as well as low digital literacy and confidence, although most would like the option of a technology-delivered program. Needs include support during PR programs (between group sessions), support after a PR program to maintain behavior change, and a complete program for those unable to attend a traditional inpatient PR program. The study also identified a difference in clinicians’ perceptions of patients’ understanding of their condition (poor) versus patients’ actual comprehension and management of their condition.

From these findings, our key considerations in designing a user-centered mPR support program will be in replicating the benefits of social support provided by the in-person group sessions, ensuring options are available for differing levels of digital literacy and confidence with technology while not providing a *second class* program for those with lower technology access is essential. Providing a program that not only considers individual technology access and literacy but also considers personal preferences and characteristics is also needed to ensure that it will be positively received by users. As indigenous populations, ethnic minority groups, and those from socioeconomically deprived groups suffer worse outcomes from COPD, ensuring an mPR program that strives for equity is essential. A strength of the survey sample was that it comprised over one-third Maori and Pacific participants. Key stakeholders highlighted the importance of considering culture and of culturally tailoring the mHealth tool.

Key considerations in terms of satisfying referring clinicians are ensuring safety can be maintained and providing appropriate programs for different groups within the population. Finding a balance between ensuring safety and maximizing access is vital to ensure that an mPR program overcomes the barriers and increases access to PR support. Although the majority of the participants in the survey and interviews had attended PR or intended to, this study has identified a range of barriers to traditional in-person PR services that align with the previous studies [9-11]. These barriers related to transport and timing can be overcome with an mHealth alternative.

It is important that these findings are interpreted in light of the study’s limitations, including the small number of participants surveyed and interviewed, the potential sampling bias in those who agreed to participate, and the patients who participated having a higher proportion accessing PR services than the general population categories. It is likely that those patients who did participate had more interest in mHealth; therefore, engagement with this type of tool may be lower in the wider population. Pretesting of an mPR program with a wider patient sample will be essential to understand the acceptability of this type of intervention.

Although there is a proliferation of technology in health care and increasingly innovative technologies being embraced for patient self-management, our study, consistent with previous studies, has shown that a digital divide exists, contributed to by differing access to data and tools as well as confidence and digital literacy to effectively use them [29-31]. Although our study demonstrated interest in using technology for PR support, the current use of many tools such as sensors and apps was low among the participants. If an mPR program is to utilize more than basic modalities, such as text messaging, then there is likely a need for these tools to be provided to patients with training and ongoing support when issues arise. This equates to additional resources and associated costs that must be considered not only in setting up an mPR program but in sustaining it.

### Conclusions

We have developed a prototype mHealth-based PR program based on the results of this mixed methods research. This includes different components suggested in the paper that will be pretested with people with chronic respiratory disorders. Feedback will be provided, and funding will be sought for the development of a full mPR program. The findings support the need for involving patients in the initial design and development of an mHealth intervention and the need to conduct a feasibility pilot study, once the intervention is developed, to try and better understand the degree of support required by health professionals and the degree of technical support required.

## Abbreviations

COPD: chronic obstructive pulmonary disease
DHB: District Health Board
GP: general practitioner
mHealth: mobile health
mPR: mobile pulmonary rehabilitation
NZ: New Zealand
PR: pulmonary rehabilitation
